# Plasma Long Chain Fatty Acids and Cognitive Function in Older Adults: A Comparison of Statistical Analyses

**DOI:** 10.1093/geroni/igab046.048

**Published:** 2021-12-17

**Authors:** Caroline Duchaine, Pierre-Hugues Carmichael, Nancy Presse, Alexandra Fiocco, Pierrette Gaudreau, Guylaine Ferland, Danielle Laurin

**Affiliations:** 1 Laval University, Quebec, Quebec, Canada; 2 Centre intégré universitaire de santé et services sociaux de la Capitale-Nationale, Quebec, Quebec, Canada; 3 Université de Sherbrooke, Sherbrooke, Quebec, Canada; 4 Ryerson University, Toronto, Ontario, Canada; 5 Université de Montréal, Montreal, Quebec, Canada

## Abstract

Omega-3 fatty acids (FAs) have been suggested as modifiable protective factors for cognitive decline because of their neuroprotective properties. However, the evidence is still inconsistent regarding types of omega-3 FAs, and the probable interrelation with other circulating long chain FAs (LCFAs). This study aimed to evaluate associations between 14 plasma LCFAs and four cognitive domains using a principal component analysis (PCA) and to compare results with those obtained using standard methods. A group of 386 healthy older adults aged 77 ± 4 years (53% women), selected from the NutCog Study, a sub-study from the Québec cohort on Nutrition and Successful Aging (NuAge), underwent a cognitive evaluation and fasting blood sampling. Verbal and non-verbal episodic memory, executive functioning, and processing speed were evaluated using validated tests. LCFAs circulating concentrations were measured by high-performance liquid chromatography using published procedures. Linear regressions adjusted for age, sex, education, and BMI were used to evaluate cross-sectional associations between LCFAs, using PCA or a more standard grouping (omega-3, omega-6, monounsaturated, and saturated LCFAs), and cognitive performance. Higher scoring on the omega-3 PCA factor and higher concentrations of total omega-3 FAs were both associated with better episodic non-verbal memory and processing speed. Higher eicosapentaenoic acid (EPA omega-3) was also associated with these two cognitive domains and with episodic verbal memory. The associations with total omega-3 FAs taken separately were of smaller magnitude than those with PCA. These results suggest that omega-3 FAs should be considered in combination with other LCFAs when evaluating the association with cognitive function.

